# Kidney outcomes for children with lupus nephritis

**DOI:** 10.1007/s00467-020-04686-1

**Published:** 2020-07-28

**Authors:** Louise Oni, Rachael D Wright, Stephen Marks, Michael W Beresford, Kjell Tullus

**Affiliations:** 1 Department of Women’s and Children’s Health, Institute of Translational Medicine, Liverpool Health Partners, University of Liverpool, Liverpool, UK; 2 Department of Paediatric Nephrology, Liverpool Health Partners, Alder Hey Children’s NHS Foundation Trust Hospital, Liverpool, UK; 3 Paediatric Nephrology, Department of Women’s and Children’s Health, Institute in the Park Alder Hey Children’s NHS Foundation Trust, Eaton Road, Liverpool, L12 2AP UK; 4 Department of Paediatric Nephrology, Great Ormond Street NHS Foundation Trust Hospital, London, UK; 5 University College London, Great Ormond Street Institute of Child Health, Biomedical Research Centre, NIHR Great Ormond Street Hospital, London, WC1N 1EH UK; 6 Department of Paediatric Rheumatology, Liverpool Health Partners, Alder Hey Children’s NHS Foundation Trust Hospital, Liverpool, UK

**Keywords:** Systemic lupus erythematosus, Lupus nephritis, SLE, Children, Prognosis, Childhood-onset SLE

## Abstract

Systemic lupus erythematosus is a rare lifelong multi-systemic autoimmune condition. Juvenile-onset SLE (JSLE) is recognized to have a more active disease course when compared with adult-onset disease and patients have a worse long-term survival. Kidney involvement occurs in over 50% of children and treatment decisions are guided by the histological classification. Several international groups have produced treatment protocols that rely on an intense period of immunosuppression to halt the acute kidney inflammatory process, followed by maintenance therapy with close observation for disease improvement and prompt evaluation of disease flares. A reduced glomerular filtration rate at presentation is predictive of later stage chronic kidney disease (CKD) in multivariate analysis. Kidney remission remains suboptimal with only 40–60% of patients achieving complete remission. Kidney flares are seen in over a third of patients. The rate of CKD 5 is reported to be up to 15% and the presence of lupus nephritis (LN) has an established link with an associated increase in mortality. In established kidney failure, transplantation seems to be the optimal kidney replacement modality for this group of patients, ideally after a period of disease quiescence. Modified outcome measures in clinical trials have demonstrated that biologic agents can be effective in this disease. Current biologic agents under investigation include obinutuzimab, belimumab, atacicept, anifrolumab, tocilizumab, eculizumab, dapirolizumab, and abatacept. Future research should focus on discovering early disease biomarkers, including surrogates for later cardiovascular disease, and evaluating biological agents as adjuncts to improve the rates of complete remission and subsequently influence the kidney outcome. The aim of this review article is to summarize the current kidney outcomes for this disease with a view to identifying key areas that may help to reduce the risk of long-term CKD.

## Introduction

Systemic lupus erythematosus is a lifelong autoimmune disease with multi-systemic features. It presents during childhood in around 15–20% of cases, although many patients with adult-onset disease report symptoms during adolescent years [1, 2]. It is a relatively rare pediatric disease with an annual incidence of 0.3–2 cases per 100,000 childhood population [3–5], with variation seen according to ethnicity and disease being more prevalent in patients of Black or Asian descent [6–8]. Juvenile-onset SLE (JSLE, also known as childhood-onset (cSLE) or pediatric SLE, pSLE) is recognized to have a more active disease course when compared with those presenting in adulthood (a-SLE), with lupus nephritis (LN) being more frequently seen [9]. Despite internationally agreed treatment protocols for LN, the rate of kidney remission remains suboptimal, the burden of disease and its treatment remains high, and long-term survival appears to have plateaued in the past few decades. The aim of this review is to summarize the prognosis of childhood LN and to discuss areas that require improvement if we are to enhance kidney outcomes.

## Lupus nephritis and the development of chronic kidney disease

**Fig. 1:**
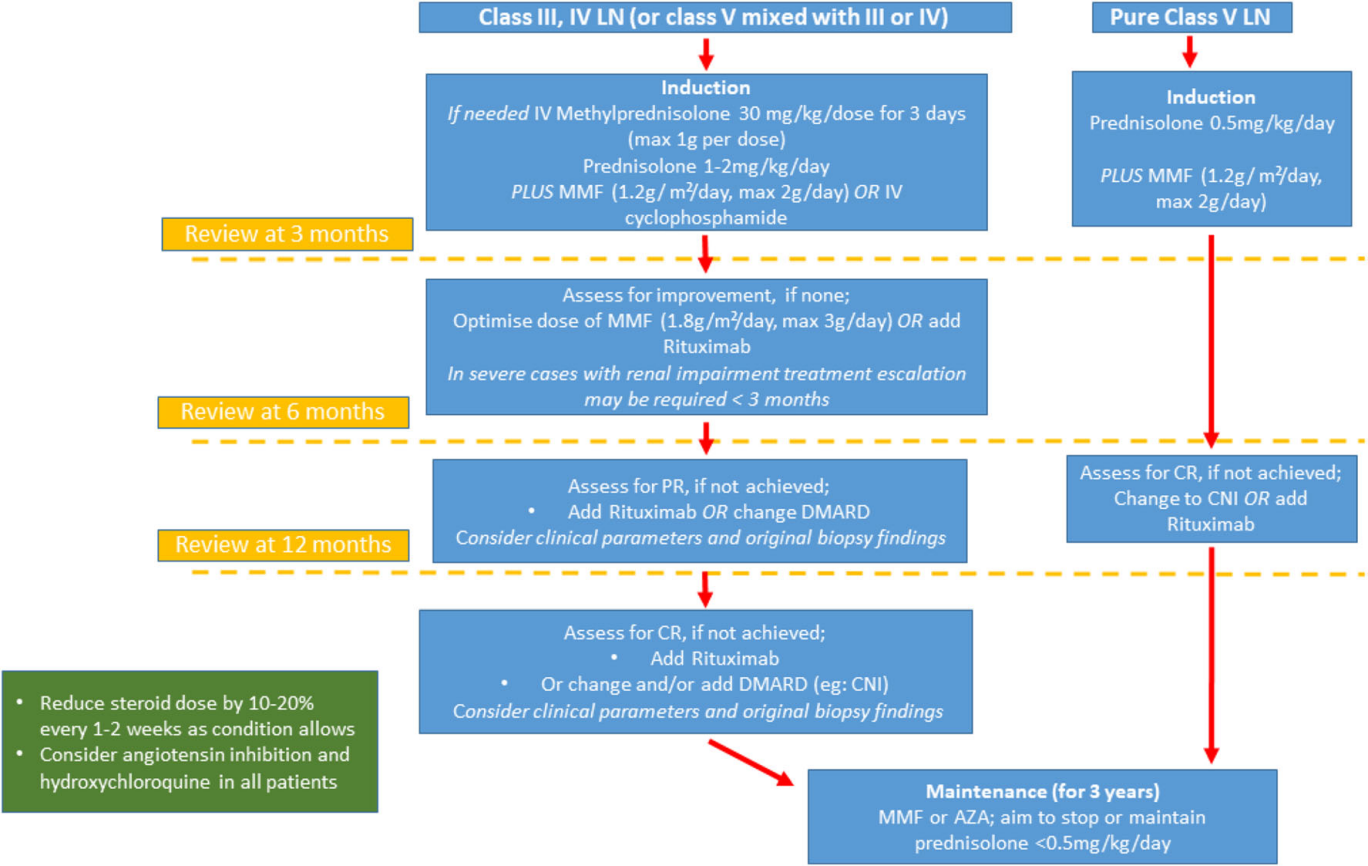
A proposed treatment protocol for the induction and maintenance management of histologically class III, IV, and V lupus nephritis in children as based on published recommendations [14–18]. CR, complete response (UPCR < 50 mg/mmol, normal kidney function); PR, partial response (> 50% reduction in proteinuria, not nephrotic, normal kidney function). UPCR, urine protein:creatinine ratio; LN, lupus nephritis; DMARD, disease modifying anti-rheumatic drug; CR, complete response; PR, partial response; MMF mycophenolate mofetil; AZA, azathioprine; IV, intravenous; CNI, calcineurin inhibitor

Lupus nephritis (LN), defined as histologically proven disease, occurs in around 50–82% of children in comparison with 20–40% of adults [10–12]. The extent of kidney involvement often dictates treatment choices in children with lupus. Treatment decisions are guided by the histological appearances that are graded according to the 2003 International Society of Nephrology/Renal Pathology Society (ISN/RPS) classification [13]. Several international groups have produced treatment protocols (Fig. 1) that are based on an intense period of immunosuppression, followed by maintenance therapy with close observation for disease improvement and prompt evaluation of disease flares [14–18]. The threshold to perform a kidney biopsy in LN is based on consensus recommendations suggesting that reproducible proteinuria of > 0.5 g/24 h (or a urine protein:creatinine ratio > 50 mg/mmol in an early morning sample) alone should prompt performing a biopsy [14, 19]. The majority of histologically proven disease is ISN/RPS class IV LN, the most active disease class associated with the worst kidney prognosis. Features of activity and chronicity seen histologically predict a poor kidney outcome [20].

Treatment response in LN has traditionally been assessed using “kidney remission” that is measured from various clinical components to create an ordinal end point (complete remission, partial remission, no remission). The precise definitions and time points vary between studies and their accuracy as a surrogate of disease improvement has been called into question. This has been partly due to the failure of being able to demonstrate proof of efficacy of a number of newer, mostly biologic, drugs in meeting the primary end points in double-blind randomized controlled trials [21]. Interestingly, more recent trials with adjusted primary outcome measures have been able to demonstrate efficacy in SLE [22]. Despite this variation, it is clear that kidney remission rates remain suboptimal with only 40–60% of patients achieving so-called complete remission (using the definition of a normal serum creatinine and urine protein ≤ 0.5 g/day) [23]. Importantly, most pediatric nephrologists strive for complete resolution of proteinuria in children (rather than accept ≤ 0.5 g/day) [24]. Low rates of complete response to treatment are likely to influence the development of chronic kidney disease (CKD) due to progressive kidney damage from ongoing inflammation. This is demonstrated in a childhood retrospective study that assessed 25 children who received a follow-up biopsy 1 year after initial LN diagnosis and a statistically significantly worse histology chronicity index score had been acquired during this short follow-up [25]. These findings show that current treatment regimens are unable to completely halt the kidney inflammatory process in the majority of patients and this contributes to damage accumulation.

In addition to initial damage, kidney flares of LN are frequently seen. A UK based adult LN cohort found that 33% of patients with histologically proven Class III or IV LN experienced a kidney flare after a mean post-induction time of 3.5 years and this seemed to be irrespective of the type of maintenance therapy used [26]. They also reported that the initial response to treatment played a significant role in the likelihood of a disease flare as 44% of partial responders experienced a subsequent kidney flare compared with only 5% of those patients who achieved an initial complete response [26]. This emphasizes the importance of aiming to achieve complete kidney remission at the outset.

**Fig. 2:**
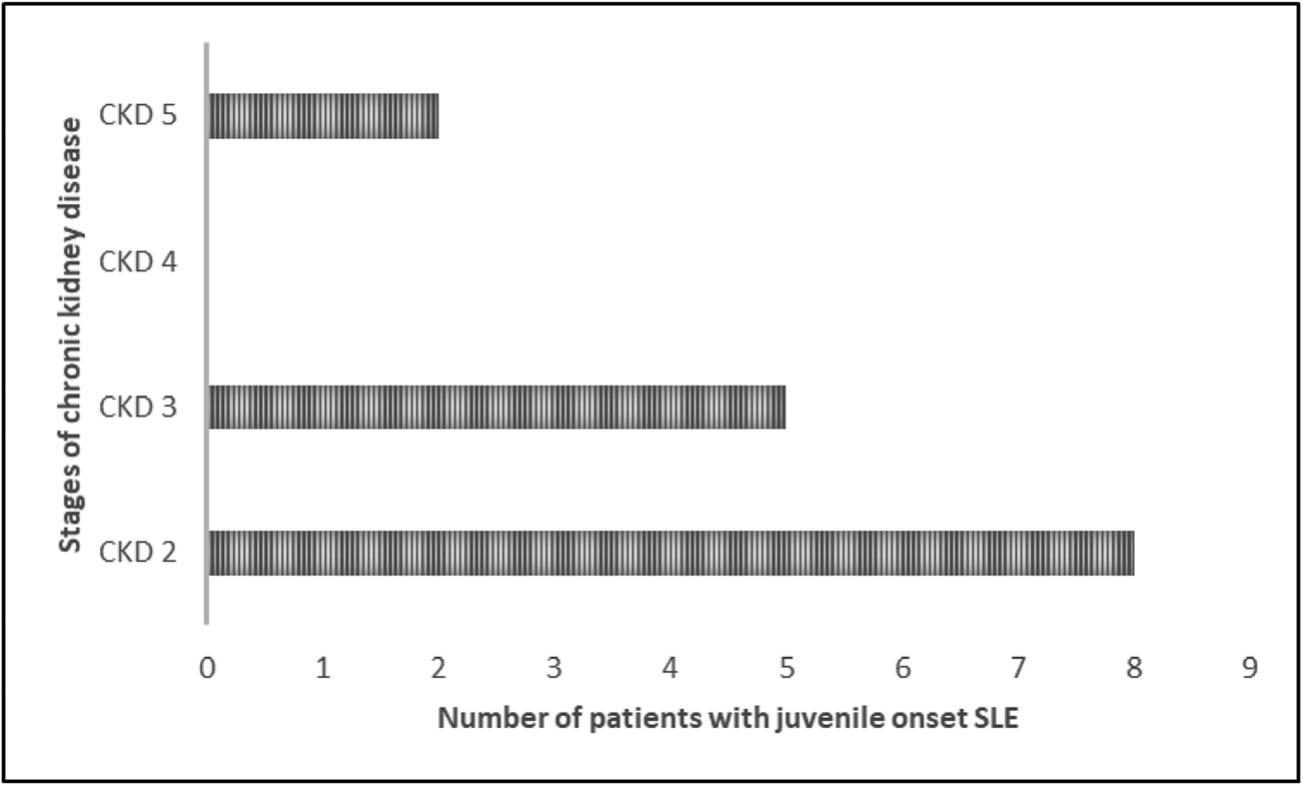
Using cohort data collected from children recruited to the UK JSLE Cohort Study [10], the incidence of chronic kidney disease (CKD) was 3.8% (15/399 children) after a median follow-up time of 6.6 years (range 0–21 years). The stages of CKD are shown demonstrating that the majority of children had CKD stage 2 (8/15; 53%, 8/399; 2% of entire cohort), followed by CKD 3 (5/15; 33%, 5/399; 1% of entire cohort), none had CKD 4 (0/15; 0%, 0/399; 0%) and 2 patients had CKD 5 (2/15; 13%, 2/399; 0.5% of entire cohort)

There are very little specific published data with regard to the risk of developing CKD prior to that requiring kidney replacement therapy (CKD stage 5/ kidney failure (KF)) in children with lupus. In adults with SLE, a large nationwide French study evaluated the burden of CKD stages 3–5 (glomerular filtration rate GFR < 60 ml/min/m^2^) [27]. It found that 6.7% of patients with LN developed CKD stage 3–5 and in the longer term CKD stage 5 was independently associated with a reduced GFR at initial presentation in multivariate analysis [27]. An initial evaluation of 399 children recruited to the UK JSLE Cohort Study [10], for the purposes of this review, demonstrated 3.8% of children had CKD stages 2–5 after an average follow-up period of 6.6 years (Fig. 2). However, the precise rates of CKD 3–5 in children from other cohorts with JSLE are unreported.

## Chronic kidney disease stage 5 in lupus nephritis

Kidney involvement in SLE has been documented for over 50 years and historically it was associated with high mortality rates. Current long-term follow-up suggests the rate of CKD 5 due to LN in childhood to be up to 15% (Table 1). A Dutch study looked at the long-term follow-up (20 years) of 111 children with JSLE and found that 67/111 (60%) had initial kidney involvement and of these 16/67 (24%) had kidney damage including 6/16 (equating to 9% of those who had initial LN) required a kidney transplant at the median age of 24 years [28]. In a cohort of 54 children from New Delhi, 48% of whom had class IV LN, 6% of children developed CKD 5 over a 10 year period [29] and an Iranian cohort of 60 children (70% had class IV LN), 15% developed CKD 5 with reported kidney survival rates of 98%, 94% and 88% for years 1, 2, and 3 after initial diagnosis, respectively [30]. A Chinese cohort in Hong Kong identified 128 children with LN and found that 51/128 (40%) presented with kidney symptoms before initial LN diagnosis, of these 4/128 (3%) had CKD 5 at follow-up (median 5.3 years [1–16.5]) [31]. Another cohort from Thailand identified 216 SLE patients of whom 180 had biopsy-proven LN, 39/180 (31%) of these patients developed kidney failure of whom 2/180 (1%) developed CKD during follow-up (median 3.9 years [9 days–19.4 years]) [32]. In adults, the CKD 5 rates are marginally lower than in children at around 6–12% over 10 years [26, 33–35]; however, in both groups, these figures may be influenced by the population being studied and the precise clinical and histological features within a given cohort.

**Table 1:** A summary of pediatric cohorts with lupus nephritis and the frequency of chronic kidney disease CKD stage 5 (or kidney failure (KF))

Study	LN patient number (*n*)	Follow-up (median [range])	Initial kidney involvement (*n*, %)	CKD stage 5 or KF (*n*, %)
Groot et al [28]	111	20 years	67/111 (60%)	16/111 (14%)
Hari et al [29]	54	10 years	No data	3/54 (6%)
Taheri et al [30]	60	3 years	No data	9/60 (15%)
Wong et al [31]	128	5.3 years [1–16.5]	51/128 (40%)	4/128 (3%)
Vachvanichsanong et al [32]	180	3.9 years [0.02–19.4]	No data	2/180 (1%)

In general, there are no specific contraindications to using any form of kidney replacement therapy in children with CKD stage 5 due to LN, although the coagulopathies commonly associated with this disease need to be considered. A study from Taiwan that included 94 adult patients with CKD 5 secondary to LN assessed the use of different kidney replacement therapy (KRT) modalities (peritoneal dialysis, PD; hemodialysis, HD; kidney transplantation, KT) and reported better survival in the KT group and thus, like many other kidney diseases, KT seems to be the optimal modality of KRT for this group of patients [36].

## Kidney transplantation in lupus nephritis

The optimal timing of performing a kidney transplant in SLE patients with CKD 5 remains uncertain and pediatric-specific data are not available. There are obvious technical risks involved in transplanting a patient with active systemic autoimmune, inflammatory disease that is associated with cytopenia, hemolysis, and pro-coagulant factors, plus active LN, could potentially develop in the new allograft [37]. The decision of when is best to transplant therefore needs to be carefully balanced against the recognized increased risks of morbidity and mortality associated with long-term dialysis. It is of the authors’ opinion that a period of 6–12 months of remission should be achieved prior to transplantation to allow for systemic disease quiescence before major surgery and to ensure that there is no chance of kidney recovery, as delayed kidney improvement is reported [38]. This may require interim dialysis. Once transplanted, there is a tendency for less SLE disease flares when compared with patients on hemodialysis (flares seen in 21% of the HD group v.s 8% in KT group, *p* = 0.06) [36] and it can be hypothesized that the efficacy of KT in LN patients may be, in part, due to post-transplant immunosuppression using combinations of disease-modifying agents with or without corticosteroids and thus the medication intensity may be playing a secondary role in keeping systemic disease quiescent and preventing further disease flares [39]. In LN transplant recipients, the outcome is good with graft survival reportedly similar to that observed in controls [40], with 5-, 10-, 15-, and 20-year graft survival reported as 81%, 79%, 57% and 51%, respectively (controls 89%, 78%, 64%, and 56%, respectively) [41].

## Disease-related damage to other organs

There is a high burden of disease-associated morbidity in JSLE [42]. In general, overall morbidity is believed to be accrued secondary to persisting disease activity, a poor response to treatment, and medication side effects. Disease damage in JSLE is common with 28% of patients in the UK JSLE Cohort Study having a Systemic Lupus International Collaborating Clinics (SLICC)/American College of Rheumatology (ACR) damage score of ≥1 after a relatively short (4.5 years) follow-up period [10] and a more recent study of the same cohort demonstrated that 85% of children still had active disease after 1 year follow-up [42]. A large Dutch study evaluating 111 patients with childhood-onset lupus followed up for a median time of 20 years showed 62% of patients had experienced damage, with damage mainly in the musculoskeletal, neuropsychiatric, and kidney systems [28, 43]. In this study, damage accrual was independently associated with disease duration, antiphospholipid antibody positivity, and hypertension. The authors also demonstrated that overall the quality of life was reduced compared with the Dutch population [28]. The extent of damage and rates of accumulated damage seem to occur regardless of age in studies comparing childhood-onset and adult-onset SLE [44].

## Treatment-induced damage

JSLE management can create a difficult conundrum of using intense immunosuppression to achieve disease remission whilst knowingly increasing the risk of treatment-associated side effects, including life-threatening infections and end-organ damage. Determining whether the etiology of organ-related damage is due to the disease itself or medications can be difficult.

LN treatment protocols heavily rely on the use of systemic corticosteroids and they are extremely useful in managing acute inflammation, yet these are typically associated with the most side effects. In the short term, corticosteroids contribute to obesity, glucose intolerance, and the development of cataracts. In the longer term, corticosteroids reduce bone density predisposing to clinical fractures and later osteoporosis, as well as worsening cardiovascular outcomes. A 12-month kidney outcome and co-morbidity study conducted by the German Society of Paediatric Nephrology in children with biopsy-proven LN noted 80% of patients had complications related to their medication use, including glucocorticoid toxicity in 42% of children and growth impairment in 78% [45]. A JSLE cohort with a longer follow-up period of 7.8 years demonstrated cataracts in 12% of children, avascular necrosis in 10%, and growth failure in 16% and these were all associated with a younger age at diagnosis [43]. Clearly, there is an urgent need for steroid-sparing protocols in children.

Hydroxychloroquine is a useful adjunct that has been shown to improve kidney outcomes [46]. It is typically very well tolerated with few side effects. It can rarely cause retinopathy and annual ophthalmology screening is therefore recommended [14, 47]. Whilst this is rare in children, in adult patients the data shows 4.3% of patients can get retinopathy although the risk rises with the duration of treatment (> 5 years) and high therapeutic levels [48].

Mycophenolate mofetil (MMF) is an important therapeutic option for induction and maintenance treatment of LN. It is usually very well tolerated although gastro-intestinal upset is frequently seen and leucopenia is not uncommon. It is associated with some serious complications including hepatic disorders, malignancy, and hypogammaglobulinemia although the incidence of these complications as a direct result of MMF in SLE is not clear and probably very rare.

Cyclophosphamide has been the standard approach to the management of LN and its introduction was associated with improved outcomes. However, it is a mutagenic agent and therefore has an associated risk of malignancy (e.g., leukemia, bladder cancer) [49]. The risk of gonadal toxicity following the use of cyclophosphamide treatment remains one of its most concerning side effects. This is dependent on both cumulative dose and pubertal status. In patients with lupus, in which associated infertility due to the disease itself is not uncommon, SLE patients exposed to high cumulative doses of cyclophosphamide have an increased risk of developing infertility and premature ovarian failure than do their counterparts treated with less toxic treatments [50].

Rituximab, a chimeric monoclonal antibody, is a useful adjunctive agent in LN. However, it commonly results in infusion reactions (seen in ~ 25%) that can be severe and life threatening. There remains concern about John Cunningham (JC) virus reactivation, leading to progressive multifocal leukoencephalopathy (PML); however, it is very rare (reported risk < 0.5%) and extremely rare in SLE, as it is reported mainly in patients with cancer who have received extensive chemotherapy [51]. Secondary drug-associated hypogammaglobulinemia due to chronic B cell depletion is being increasingly reported and predisposes individuals to infection. It is seen in around 4% of patients and may require lifelong immunoglobulin replacement [52].

## Long-term associated cardiovascular disease

Long-term associated cardiovascular morbidity is extremely common in patients with lupus, due in part at least to the chronic inflammatory state, and this risk is increased in the presence of nephritis and contributes significantly to mortality [52]. In a study by Groot et al., reporting the long-term follow-up of childhood-onset SLE, cardiovascular complications such as myocardial infarctions began occurring after only a 5-year disease duration and these occurred young, with the median age of having a cerebrovascular accident being 20 years and a myocardial infarction at a median age of 39 years [28]. Care models based on actively screening for cardiovascular complications can improve detection and may provide a step towards preventing long-term morbidity in this cohort [53].

## Lupus-associated mortality in children

The intense immunosuppressive regimens utilized to control active LN increases the risk of serious infection, so the mortality associated with JSLE is usually due to infection on a background of very active disease [6] and it is a leading cause of death in SLE patients worldwide. In a UK comparison study, children with SLE had an increased standardized mortality ratio of 18.3 compared with a ratio of 3.1 reported in adult-onset SLE [53]. In India, Aggarwal et al. studied 273 children with SLE (92% girls, median diagnosis age 14 years) over a follow-up period of 3.5 years and reported infections in 72 children (26%), with over half of these being serious, and fourteen children (6%) died [54]. The 5-year mortality rate for childhood lupus is reported to be between 4 and 23%, with rates varying according to the age of disease onset and the population being studied [55]. Younger children have the highest mortality rates as demonstrated within JSLE cohorts, where the effect of age can be clearly seen. In a Brazilian study, children with very early-onset disease (diagnosed age < 6 years) were predisposed to an increased risk of death (15%) compared with those of middle childhood age (school age ≥ 6 and < 12 years) who had a 10% risk of mortality, and adolescents (aged ≥ 12 and < 18 years) who had a 6% risk of mortality [6]. Death in JSLE often occurs during the acute phase as reported in a study from Brazil (847 children), where 33/69 (48%) of deaths occurred within the first 2 years after diagnosis [6].

Patient survival improved historically until the last few decades when it appears to have plateaued. A worldwide systematic review of SLE survival rates in both adults and children from the 1950s through to 2016, included 46,317 adult patients (from 125 adult studies) and 6,862 children (from 51 pediatric studies) from both high-income countries (HIC) and low-/middle-income countries (LMIC) [9]. The authors found that after a period of major improvement, survival in SLE has plateaued since the mid-1990s. Differences were seen when evaluating survival in children according to the nation’s income with recent survival rates only improving in the children from HIC’s, with 5-year and 10-year survival rates from 2008 to 2016 found to be 0.99 and 0.97 for children in HIC and only 0.85 and 0.79 for children in LMIC. This difference was not observed among adults. International access to pediatric care may explain some of these findings. It is not yet clear, whether medications introduced in the last decade, such as mycophenolate mofetil and rituximab, would have enhanced survival at the population level.

The presence of LN has an established link with increased associated mortality rates in SLE. A single-centre study of JSLE patients in India reported a mortality rate of 20% with the major predictor of mortality being the serum creatinine at the time of clinical presentation [56]. In a 23-year follow-up study from Iran, 11% of patients (20/180) with JSLE and 9% of patients (35/394) with adult SLE (a-SLE) died and the authors found that the mortality rate was not significantly different between the two groups (*p* = 0.4) but the main causes of death did differ between the groups, with more nephritis (50% in JSLE vs. 29% in a-SLE) and more infections (40% in JSLE vs. 29% in a-SLE) seen in the children. By multivariate analysis, seizure, proteinuria, and nephritis in JSLE had a negative prognostic effect on survival [57] and as such the importance of kidney disease as a primary cause and main predictor of death in SLE is a consistent finding in the literature [58].

## Improving kidney outcomes in JSLE

Modifiable risk factors associated with a worse kidney prognosis that could potentially be influenced include the early recognition of active kidney disease, as delays can result in damage accrual. Recent international efforts in this regard indicate that the use of a panel of novel urinary biomarkers is superior to current clinical markers and integrating these into practice may enhance earlier detection and intensification of management [59]. Detailed discussion of biomarkers is beyond the scope of this paper. In addition to novel biomarkers, standardizing aspects of care may help, including when to perform a kidney biopsy and histological interpretation. Recently published, international consensus recommendations for childhood LN include these aspects and integration of these recommendations into practice should be a clinical priority [14]. The modified histological classification scores for LN incorporating active/chronic lesions seem to be more robust in predicting outcomes [60] and allow additional information on the kidney activity and chronicity status of each patient.

As this is such a heterogeneous autoimmune disease, clinical trials targeting specific biological pathways, may provide improved rates of complete response in certain patients. Examples of novel agents in the process of being evaluated for the treatment of LN are summarized in Table 2. A more personalized treat-to-target approach may explore individual immune profiling for patients who fail to achieve complete remission in order to identify predominant immune components (such as B cells, complement products, specific cytokines) that are able to act as treatment targets. This will require further scientific understanding of the biological pathways involved in active LN, and a clinically robust method of immune cell profiling that is designed and based around existing biological therapies. The identification of individual genetic risk factors may also enhance this process and perhaps pro-active targeting of high-risk groups such as patients of African, Asian, or Hispanic descent who have worsened outcomes compared with Caucasians, who may benefit from a more intensive approach to treatment [61]. The addition of agents to the current induction treatment may also prove to be superior, such as adjunctive early B cell targeting or adjunctive calcineurin inhibitor [62].

**Table 2:** Promising novel agents being evaluated for use in LN and their stage in drug development

Biological target	Drug name	Studies	Phase in drug development
B cell depletion	Obinutuzumab	[63]	Phase II
B cell growth and survival inhibitor	Belimumab	[64]	Food and Drug Administration (FDA), European Medicines Agency (EMA), and National Institute for Health and Clinical Excellence (NICE) approval for use in adults with LN
	Atacicept	[65]	Phase II
Type 1 interferon	Anifrolumab	[66]	Phase I. Phase II/III in planning
Interleukin-6	Tocilizumab	[67]	Phase I
Complement	Eculizumab	[68]	Phase I
T cell anti-CD40L	Dapirolizumab	[69]	Phase II
T cell costimulatory pathway	Abatacept	[70]	Phase III

As with any long-term disease, adherence to medications remains a concern. A nationwide longitudinal study in Germany on adults with SLE found that only 63% of patients self-reported high adherence [71] and another study on childhood rheumatic diseases described worse adherence in those patients required to take > 3 daily medications [72]. Rationalizing medications when possible and regular discussion to understand the complexity of adherence is therefore important.

On an international basis, the more challenging factors to address are the international variation in outcomes with regard to children with JSLE from low-/middle-income countries and this relies on mass changes in social, political, and health care agendas to provide equitable access to pediatric care across the world.

## Conclusions

JSLE is a severe, lifelong condition and the overall mortality remains high across the world. Kidney involvement is independently linked to an increased risk of mortality and more damage accrual. The prompt recognition and management of LN is essential. Despite international consensus protocols, the evidence supporting LN management remains poor and the kidney outcomes suboptimal. There is an urgent need to improve many aspects regarding clinical care and our understanding associated with this disease, in particular the evaluation of novel therapies as adjuncts to current treatment to improve outcomes.
